# Defining Soilborne Pathogen Complexes Provides a New Foundation for the Effective Management of Faba Bean Root Diseases in Ethiopia

**DOI:** 10.3390/pathogens14070695

**Published:** 2025-07-14

**Authors:** Solomon Yilma, Berhanu Bekele, Joop Van Leur, Ming Pei You, Seid-Ahmed Kemal, Danièle Giblot-Ducray, Kelly Hill, Thangavel Selvaraji, Alemu Lencho, Lemma Driba, Martin J. Barbetti

**Affiliations:** 1 Ethiopian Institute of Agricultural Research, Ambo Agricultural Research Center, Ambo P.O. Box 37, Ethiopia; solomonyilma509@gmail.com (S.Y.); b_bekele2000@yahoo.com (B.B.);; 2 Department of Plant Science, School of Agriculture, Ambo University, Ambo, Ethiopia; tselvaraj1956@gmail.com (T.S.); alemulencho@gmail.com (A.L.); 3 New South Wales Department of Primary Industries, Tamworth Agricultural Institute, Tamworth, NSW 2340, Australia; 4 School of Agriculture and Environment and the UWA Institute of Agriculture, The University of Western Australia, Crawley, WA 6151, Australia; 5 International Center for Agricultural Research in the Dry Areas, Station Exp. INRA-Quich, Rabat Instituts, Rabat 10112, Morocco; 6 South Australian Research and Development Institute (SARDI) Plant Research Centre, Urrbrae, SA 5064, Australia; daniele.giblot-ducray@sa.gov.au (D.G.-D.); kelly.hill3@sa.gov.au (K.H.); 7 School of Agriculture, Food and Wine, The University of Adelaide, Urrbrae, SA 5064, Australia

**Keywords:** soilborne pathogen complexes, new host records, phylogenetic analysis, phylogenetic analysis/determination, HTS metabarcoding, pathogen complexes, faba bean, root diseases

## Abstract

Soilborne diseases cause losses of 45–70% in faba bean in Ethiopia. Studies were undertaken to define soilborne pathogens and their complexes in Ethiopia. First, the severity of root rot was assessed in 150 field sites across seven Ethiopian regions. Soil samples were collected, and the DNA of 29 pests and pathogens was quantified using a commercial quantitative PCR (qPCR) soil testing service. There was a very high incidence rate of *Macrophomina phaseolina*, as well as *Pythium* clades F and I. The other detected species in order of incidence included *Fusarium redolens*, *Rhizoctonia solani*, *Aphanomyces euteiches*, *Phytophthora megasperma*, *Sclerotinia sclerotiorum* and *S. minor*, and *Verticillium dahliae,* as well as low levels of *Thielaviopsis basicola.* Five anastomosis groups (AG) of *R. solani*, namely AG2.1, AG2.2, AG3, AG4, and AG5, were detected, of which AG2.2 and AG4 were most prevalent. We believe this is the first report of occurrence for Ethiopia of *A. euteiches*, *Ph. megasperma*, *T. basicola,* and the five AGs for *R. solani*. There were very high incidence rates of the foliar pathogens *Botrytis cinerea*, *B. fabae, Didymella pinodes*, and *Phoma pinodella* and of the nematode *Pratylenchus thornei,* followed by *P. neglectus* and *P. penetrans*. The root rot severity and distribution varied significantly across regions, as well as with soil types, soil pH, and soil drainage. Subsequently, metabarcoding of the soil DNA was undertaken using three primer pairs targeting fungi (ITS2), *Fusarium* species (TEF1 α), and Oomycetes (ITS1Oo). The ITS2 and TEF1α primers emphasized *F. oxysporum* as the most abundant soilborne fungal pathogen and highlighted *F. ananatum*, *F. brachygibbosum*, *F. brevicaudatum*, *F. clavum*, *F. flagelliforme*, *F. keratoplasticum*, *F. napiforme*, *F. nelsonii*, *F. neocosmosporiellum*, *F. torulosum*, and *F. vanettenii* as first reports of occurrence for Ethiopia. The ITS1Oo primer confirmed *Pythium* spp. as the most prevalent of all Oomycetes.

## 1. Introduction

Faba bean (*Vicia faba*) is an early domesticated crop that remains an important food and feed crop in many countries [1]. It is mainly grown in Australia, Europe, Egypt, Ethiopia, and China [2], with Ethiopia the second most important producing country after China. Ethiopia is considered a secondary center of faba bean diversity [3] and grows more than half a million ha as a rainfed crop [4]. Faba bean is highly nutritious as human food and plays an important role in the Ethiopian economy, producing the highest percentage of protein (20–41%) per unit of land area [5] when compared with lentil (*Lens culinaris*), common bean (*Phaseolus vulgaris*), chickpea (*Cicer arietinum*), and pea (*Pisum sativum*).

Despite the development and registration of high-yielding varieties of the crop and its wide economic importance, the average national yield of faba bean in Ethiopia for small-holder farmers is 2.1 t/ha, with the productivity in some regions being much lower than this national average [4]. This is due to faba bean’s vulnerability to biotic and abiotic stresses [6]. Faba bean crops are generally grown continuously or with a simple and repeated rotation in the same field, which fosters the development of soilborne diseases; an inoculum of soilborne pathogens then accumulates [7] and persists, as these pathogens often have a wide host range along with a range of effective mechanisms for survival, even in the absence of a susceptible host. This makes the development and application of management strategies for soilborne diseases in faba bean not only challenging but also critically dependent on the ability to determine the identity and incidence of soilborne root rot pathogens.

A range of soilborne pathogens are known to cause root rot in faba bean [8], including *Fusarium solani*, *Rhizoctonia solani*, *Sclerotium rolfsii*, *F. oxysporum*, *F. avenaceum*, and *Pythium* spp. [9,10,11,12]. These pathogens are common throughout the faba bean growing zones of Ethiopia, resulting in yield losses of 45–70% under severe conditions [8], even reaching 100% for the more susceptible cultivars [13], especially in high-moisture black soils [14,15]. However, while one or more soilborne pathogens have been reported to be associated with root disease in faba bean, the overall situation regarding soilborne pathogens of faba bean in Ethiopia remains undefined, particularly in relation to soilborne pathogen complexes.

The identification of soilborne pathogens using cultural and morphological characteristics is not only challenging but frequently fails to define the pathogen components involved in complexes containing multiple pathogens, as so often occurs with root disease. This makes it challenging to manage soilborne disease complexes [16], especially as fungicides are generally inadequate in such situations [17,18,19]. The ability to reliably define the pathogens involved in soilborne disease complexes is foundational to being able to predict and manage them [20]. In recent years, there have been significant advances in utilizing molecular approaches to define and understand the diversity of soilborne pathogen communities. These include pathogen-specific quantitative polymerase chain reaction (qPCR)-based methods [21] and high-throughput sequencing-based methods, in particular DNA metabarcoding for a more general approach to pathogen surveillance.

As faba bean crops had not previously been comprehensively surveyed for the incidence and severity of root disease in Ethiopia, this paper reports the outcomes from a comprehensive root disease survey undertaken across the different faba bean cropping zones. Additionally, as soilborne pathogens associated with root disease of faba bean have never been defined, 150 soil samples from across the surveyed fields were sent for a further analysis to the commercial quantitative PCR (qPCR) soil testing service developed by the South Australian Research Development Institute (SARDI, Adelaide, Australia) [21]. There, qPCR testing was used to specifically identify and quantify the relative DNA amounts of 29 pathogens or pathogen groups. DNA metabarcoding [22] targeting three gene regions was undertaken to provide taxonomic information for the three overall groups of organisms of interest, namely general fungi, *Fusarium* spp., and Oomycetes.

## 2. Materials and Methods

### 2.1. Description of the Study Area

**Figure 1:**
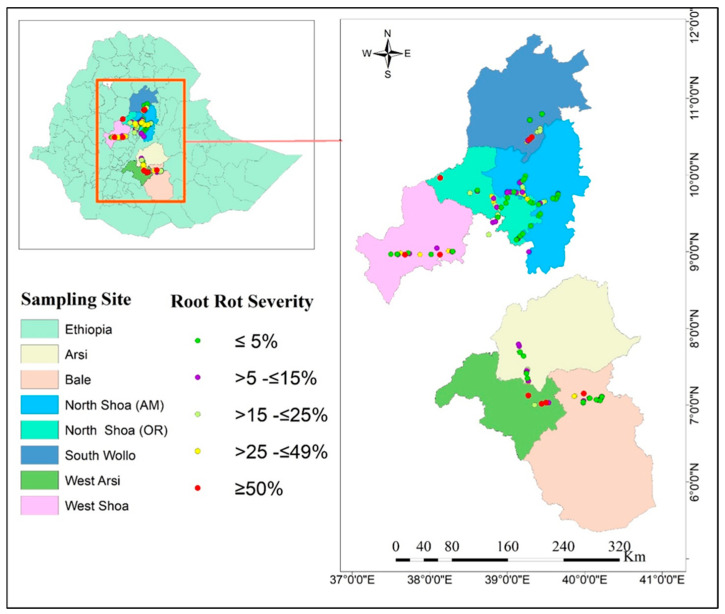
Map of the faba bean root rot study area within Ethiopia, showing different geographical zones and sites sampled in the root diseases survey.

The survey of faba bean root diseases and soil sampling was conducted in 2021 during the main cropping season across 150 random faba bean and pulse cropping zones of the Amhara (South Wollo, North Shoa) and Oromia (West Shoa, Arsi, West Arsi, Bale, and North Shoa) regions, which include mid to extreme highlands across the geographical zones of Ethiopia. These zones typically have a bimodal rainfall distribution and high-altitude climatic conditions (Table 1). A map highlighting the study areas and sites sampled is shown in Figure 1. Of the 150 faba bean and pulse crop sites, 126 were associated with faba bean crops, 18 with field pea (*Pisum sativum*) crops, and 6 with lentil (*Lens culinaris*) crops.

### 2.2. Assessment of Faba Bean Root Rot

The faba bean and pulse fields in each zone were randomly chosen at approximately 5–10 km intervals following main, secondary, and other rural roads. At each sampling site, the root rot disease severity was assessed at 5 locations 20–50 m apart (depending upon the size of the field) within each field across two diagonal transects (i.e., ‘X’). At each sample point, plants within a 1 m^2^ quadrant were counted, assessed, and recorded as either diseased (or infected) or healthy (or non-infected). These plants were subsequently assessed for their level of root disease based on a 0–5 scale as described by Davis et al. [23], where 0 = no obvious symptoms, 1 = a few discolored lesions on roots, 2 = minor discoloration covering < 90% of the root system, 3 = >90% of the root system shows brown or yellow discoloration but no symptoms on the epicotyl or hypocotyl, 4 = all of the root system shows brown discoloration or soft rot with the epicotyl and hypocotyl being brown and shriveled, and 5 = the plant is dead. Then, for each sample, the root disease Percentage Severity Index (PSI) value was calculated, as follows:

$$PSI=\frac{Sum\;of\;numerical\;score\times 100}{Number\;of\;plant\;scored\;\times Maximum\;score\;on\;scale}$$

Data on the geographical information (latitude, longitude and altitude) of each field were recorded using GPS software installed on a Samsung Galaxy Tab A6. Additional data on crop variables such as the growth stage, improved or local variety, precursor crop, soil type, method of planting (raised-bed or flat-bed type), and planting date were recorded.

### 2.3. Collection of Soil Samples from Farmer Fields

Soil samples were taken from the same 150 faba bean pulse fields. Prior to sampling any field, all sampling equipment was surface sterilized with 70% ethanol to prevent cross-contamination. In each field, two circles 6 m in diameter were marked out, within which smaller circles of 3 m in diameter were marked out. Then, using the methodology of Huising et al. [24], 13 sampling points were arbitrarily chosen to take a 10-cm-diameter soil core down to a depth of 20 cm, from which a 250 g subsample of the soil was extracted. The total of 13 cores taken at each location were combined, from which a 1 kg composite subsample was taken, sealed in a plastic bag, and kept out of sunlight [25]. The soil samples were brought to the Ambo Agricultural Research Center in an ice box, where they were maintained thereafter at 4 °C. Subsequently, the soil samples were air-dried and then air-freighted to SARDI (Adelaide, Australia), where they were maintained in a drying oven until being processed.

### 2.4. Extraction, Identification, and Quantification of DNA from Soils

DNA extraction from the soil samples was undertaken by the SARDI commercial quantitative PCR (qPCR) soil testing service [21]. The soil DNA was tested using qPCR to specifically identify and quantify the DNA of 29 root and foliar pathogens. This method has been utilized earlier for Australian surveys of root disease pathogens on forage legumes [26].

### 2.5. High-Throughput Sequencing (HTS) Metabarcoding Studies of Samples

High-throughput sequencing (HTS) metabarcoding (i.e., targeted amplification of one or more gene regions known to provide taxonomic information for the group of organisms of interest) was also carried out on the same soil DNA that had undergone quantitative PCR (qPCR) soil testing. The regions chosen were selected to provide enough genetic diversity to distinguish taxa at the required level for biologically relevant information. ITS2 (ITS86/ITS4) [27,28], ITS1 (biased to Oomycetes using ITSOo/ITS7) [29,30], and TEF1α primers (biased to *Fusarium* using TEF1_198f/TEF1_593r) [31], coupled to Illumina overhangs, were used to generate amplicons. Barcoded samples were further generated using unique dual indices (IDT) and pooled prior to sequencing.

Sequencing was carried out on the Illumina MiSeq platform by the Australian Genome Research Facility (AGRF). The ITS2 amplicons were sequenced using the 2 × 300 bp MiSeq reagent kit v3, while the TEF1α and ITSOo amplicons were sequenced using the 2 × 250 bp MiSeq reagent kit v2.

### 2.6. Bioinformatics and Statistical Analysis

The survey data and results of the quantitative PCR (qPCR) soil tests were analyzed using R-software version number 4.0.2 [32]. The DNA concentrations of the respective pathogen, soil type, soil pH, agro-ecology, and PSI values for root disease were subjected to an analysis of variance (ANOVA), while the Tukey test of the least significant difference (LSD) was used for mean separation at the 5% level of significance.

The Illumina sequences were quality-filtered and trimmed using the Quantitative Insights into Microbial Ecology 2 (QIIME2) software package (v2019.4) [33] and the Dada2 [34] algorithm was used for denoising the data, including checking and removing chimeric sequences. The resulting sequences, termed amplicon sequence variants (ASVs), were assigned taxonomy using the sklearn plugin [35] and a pre-trained classifier using the Qiime release UNITE database (version 10.05.2021) for ITS2. The Vsearch algorithm [36] and a database generated from NCBI downloaded in 2022 were used for ITSOo sequences, using the following search terms: ((“Fungi” [Organism] OR (“Fungi” [Organism] OR “Fungi” [All Fields]) OR (“Nematoda” [Organism] OR (“Nematoda” [Organism] OR “Nematode” [All Fields]) OR Protist [All Fields] OR Oomycete [All Fields])) AND ((internal transcribed spacer 1 [All Fields] NOT (UNVERIFIED [All Fields] OR Uncultured [All Fields]) AND (“100” [SLEN]: “10000” [SLEN])). Again, the Vsearch algorithm [36] and a database generated from NCBI also downloaded in 2022 were used for TEFα sequences, using the following search term: {(“Fungi” [Organism] OR (“Fungi” [Organism] OR (“Fungi” [Organism] OR (“Fungi” [Organism] OR “Fungi” [All Fields] OR (“Nematoda” [Organism] OR (“Nematoda” [Organism] OR Nematode [All Fields] OR “Protist”[All Fields])) AND ((translation elongation factor 1 [All Fields] OR TEF1 [All Fields]) NOT (UNVERIFIED [All Fields] OR Uncultured [All Fields]) AND (“100” [SLEN]: “1800” [SLEN])}. The sequences were further filtered using the decontam package [37] and visualized using phyloseq [38] and ggplot2 [39] in Rstudio [40].

## 3. Results

### 3.1. Soil Typology Across the Geographical Zones

**Figure 2:**
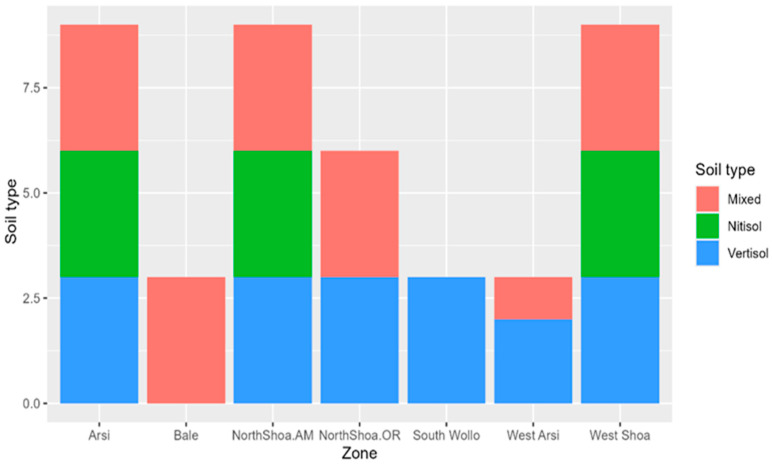
Prevailing soil type distribution across the sampled geographical zones.

The farms situated in the different geographical zones had varying soil types, including 55 farms with vertisol (black soil), 63 farms having mixed soil (humus rich soil), and 32 farms with nitisols (reddish soil) across the different geographical zones. The dominant soil type in the study area was vertisol, which was present in most zones except Bale, where mixed soil was the only soil type. Vertisol was the only soil type in South Wollo. The zones lacking nitisol soils included North Shoa Oromia, Bale South Wollo, and West Arsi (Figure 2).

### 3.2. Soilborne Pathogens Detected Using qPCR

From the 150 soil samples analyzed using the quantitative PCR (qPCR) soil testing service, 26 of the 29 root and foliar disease pathogens tested for were detected. Table 2 shows the detected pathogens, their relative incidence rates, and their total DNA concentrations per gram of soil. Across the 150 samples, consisting of 126 that were associated with faba bean cropping, 18 with field pea cropping, and 6 with lentil cropping, there were generally overall similar trends in terms of pathogen incidence rates across the three different host species (Table 2). A wide range of fungal and Oomycete soilborne pathogens, many of which co-occur in pathogen complexes, were detected, including very high incidence rates of *Macrophomina phaseolina* and *Pythium clades* F and I, followed by *Fusarium redolens*, *Rhizoctonia solani,* and *Aphanomyces euteiches.* There were low incidence rates of *Phytophthora megasperma*, *Sclerotinia sclerotiorum*, *S. minor*, and *Verticillium dahliae*, and very low incidence rates of *Thielaviopsis basicola*, *Phytophthora medicaginis*, and *Phoma rabiei*. Five anastomosis groups of *R. solani* were identified, namely AG2.1, AG2.2, AG3, AG4, and AG5, of which AG2.2 and AG4 were most prevalent. Of the foliar fungal pathogens, there were very high incidence rates of *Botrytis cinerea*, *B. fabae, Didymella pinodes*, and *Phoma pinodella*. Of the nematodes, *Pratylenchus thornei* showed very high incidence rates. Low incidence rates of *P. neglectus* and *P. penetrans* and very low incidence rates of *P. quasitereoides, Meloidogyne javanica*, *Meloidogyne incognita*, and *Meloidogyne arenaria* were recorded (Table 2). *R. solani AG8*, *Phoma koolunga,* and *Ph. drechsleri* were not detected in any of the samples. The percentage severity indices and corresponding DNA concentrations of the respective pathogens are displayed in Table 3. While overall the severity of the observed symptoms is likely due to various combinations of the pathogens detected and their interactions, it did appear that for some pathogens, a high PSI (>50) score was associated with a high DNA concentration, such as for *Pythium* clade F (417 pg DNA/g) and *Pythium* clade I (1651 pg DNA/g) (Table 3). A similar pattern was recorded for *V. dahliae*, *Ph. megasperma*, and *B. fabae.* In contrast, for pathogens such as *A. euteiches*, the maximum value of DNA corresponded with the lowest PSI score (1–5). However, for many of the pathogens, there was no association of the PSI score with the amount of DNA.

### 3.3. Distribution of Soilborne Pathogens in Relation to Geographical Zones

**Figure 3:**
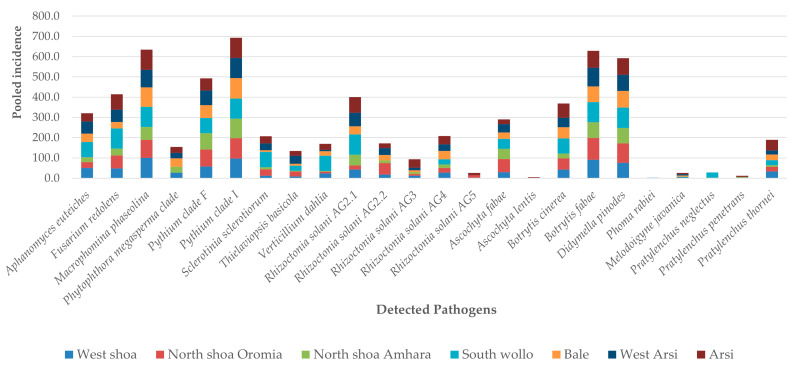
Distribution of identified soilborne pathogens across the different geographical zones. Note: Their ‘pooled incidence across different zones’ represents the total summation of the individual incidence in each zone for each pathogen.

The relative distributions of soilborne pathogens and nematodes across the different regional zones and how the soil type and geographical zone impact them are shown in Figure 1 and Figure 3 and Table 4, respectively. The soil pH and PSI levels were highly influenced by the geographical zone (Table 4). However, 15 pathogens were not affected by the geographical zone, including *M. phaseolina*, *R. solani* AG4 and AG5, *Pythium* clade F, *P. neglectus*, *P. penetrans,* and *P. quasitereoides* (Table 4), while 13 other pathogens, including *D. pinodes*, *B. fabae*, *R. solani* AG4, and *Pythium* clade I, were found in all geographical zones (Figure 3). *A. euteiches*, *M. phaseolina*, *Pythium* clade I, *Pythium* clade F, and *M. javanica* were more abundant in the West Shoa zone than other zones, while *A. euteiches* was significantly higher in West Arsi and West Shoa. *S. sclerotiorum* and *S. minor*, *R. solani* AG2-1, *R. solani* AG2-2, *R. solani* AG4, and *R. solani* AG5 were not distributed significantly differently across the different geographical zones, although there was a tendency for the DNA values of *R. solani* AG2-2 to be higher in the South Wollo, West Arsi, and Bale zones (Table 5). *V. dahliae*, *P. neglectus*, *P. penetrans,* and *P. thornei* were completely absent in the Bale zone. The plant-parasitic nematodes generally showed low levels across all zones except for *M. javanica*, *M. incognita*, and *M. arenaria.* The soil pH and root rot severity were significantly higher in the West Shoa zone but lower in the South Wollo and Bale geographical zones (Table 5).

### 3.4. Distribution of Soilborne Pathogens and Percentage Severity Index (PSI) Scores in Relation to Soil Type

The soil pH and PSI were highly influenced by the soil type, with both being significantly higher in the vertisol than the other two soils (Table 4). All three soil types contained root-rot-causing pathogens, as well as other soil-inhabiting fungi or plant-parasitic nematodes (Table 4 and Table 6). The soilborne pathogens *A. euteiches*, *F. redolens*, *P. megasperma*, *Pythium* clade F, *Pythium* clade I, *S. sclerotiorum*, *S. minor*, *T. basicola*, *V. dahliae,* and *R. solani* AG2.2 showed the highest levels in the vertisol but the lowest levels in the mixed and nitisol soils (Table 6). *B. fabae* was less frequent in vertisol soils than the other two soil types, while the levels of *M. javanica*, *M. incognita*, *M. arenaria,* and *P. thornei* were significantly higher in the vertisol soils (Table 6). Other pathogens such as *M. phaseolina*; *R. solani* AC2.1, AG4 and AG5; *Pythium* clade F; *P. neglectus*; *P. penetrans;* and *P. quasitereoides* were not affected by the soil type, with some such as *D. pinodes*, *B. fabae*, *R. solani* AG.4, and *Pythium* clade I being commonly found across all soil types. In general, the soilborne fungal and nematode levels were similar across both mixed and nitisol soils.

### 3.5. Effect of Soil Drainage on Distribution of Some Selected Soilborne Pathogens

As vertisols are the most problematic soils in Ethiopia due to inherent waterlogging and poor drainage, we compared vertisol soils versus combinations of other soil types and listed the average root rot incidence values for several key pathogens for drainage (raised beds) versus no drainage. The beneficial effect of drainage on lowering the root rot incidence is clear for some pathogens such as *Pythium* clade I, *R. solani* AG2.2, and *R. solani* AG4 in vertisol soils but not in other soil types (Table 7).

### 3.6. Metabarcoding of Soilborne Pathogens—Taxonomic Overview

Over the 150 samples, 11.6 million sequences were generated using the ITS2 primers [36]. After quality filtering and merging, approximately 6.6 million reads were retained, with a mean read length of 276 bp. This gave an average sequencing depth of approximately 44,000 sequences per sample. Unassigned taxa, taxa with <10 reads, and any sequences assigned to Viridiplantae were removed prior to a further analysis. The TEF1α primer libraries produced 5.5 million sequences, with 2.5 million retained after quality filtering and merging, with a mean read length of 285 bp. This gave an average sequencing depth of approximately 16,000 sequences per sample.

**Figure 4:**
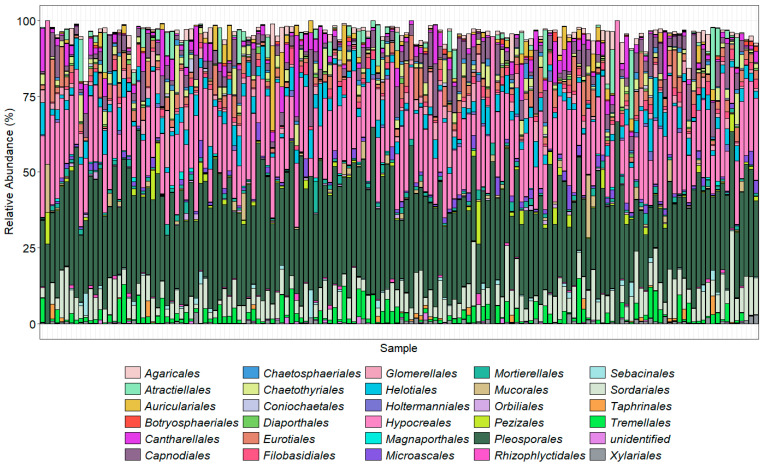
Using ITS2 primers, the top 30 most abundant orders in each sample as a percentage of that sample. Colors generally follow the order of the legend; for example, Agaricales is at the top of the stacked column and Xylariales at the bottom.

**Figure 5:**
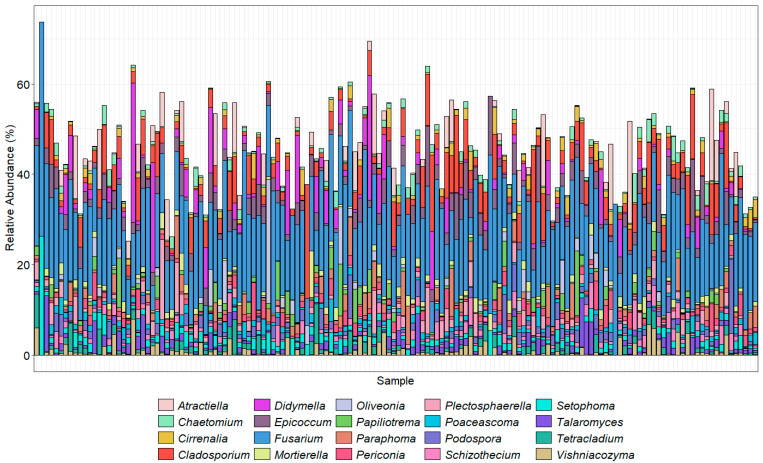
The top 20 most abundant genera found in each sample using ITS2 primers. Sample taxonomy was assigned using the UNITE database (version 10.05.2021). Colors generally follow the order of the legend, with *A. proliferata* at the top of the stacked column where present and *Zopfiella marina* at the bottom.

The bar charts display the predominant taxa in the samples as a percentage of the total for the top 30 most abundant orders (Figure 4) and the top 20 most abundant genera (Figure 5) found using the ITS2 primers. The TEF1α primers were designed to bias the amplification for *Fusarium* spp.; however, they are degenerate primers and amplify other fungal taxa with varied efficiencies. Here, we show the *Fusarium* spp. identified using our taxonomic assignment methods. The taxonomic assignment was further clarified where possible by alignment of these sequences with those downloaded from *Fusarium*-ID v.3.0 [40]. Some sequences, however, were not present in this database. Neither the ITS2 nor TEF1α gene regions provide enough resolution to confidently separate the *formae speciales.* Amplicons prepared with the primer ITS1Oo [41], which generated 7.8 million sequences that were reduced to 3.2 million (with an average 21,600 sequence per sample), with a mean read length of 245 bp.

**Figure 6:**
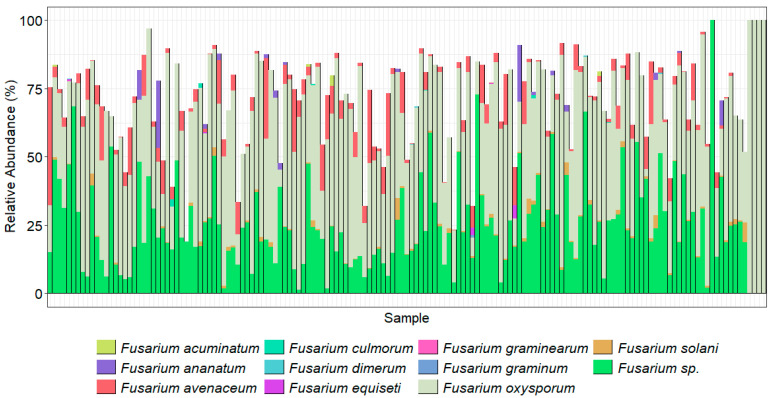
*Fusarium* species present in each sample as amplified by TEF1α primers. Taxonomy was assigned using the TEF1α gene region, Vsearch assignment method, and a database generated from GenBank (downloaded on 29 August 2022). Species were confirmed using alignments with *Fusarium*-ID v 3.0.

The orders with the highest proportions of sequence reads identified using the ITS2 primer pair were Pleosporales (*Phoma* and *Alternaria*), Hypocreales (*Fusarium*, *Claviceps*, *Erysiphe*, *Phyllactinia*, *Podosphaera*, *Sphaerotheca,* and *Uncinula*, which includes the causal agents of powdery mildew, with many specialized races of fungal species in the genera), and Sordariales, which included species of *Ophiostoma* (the cause of Dutch elm disease), *Gnomonia* (leaf spots), *Diaporthe* (stem and leaf blights), Tremellales (yeast fungi), Cantharellales (*Rhizoctonia*), and Helotiales (*Botrytis cinerea*) that were abundantly present in the majority of the samples. As the ITS2 region is not always adequate alone to distinguish down to species level, the results were analyzed at the genus level. Of the top 20 genera found across the 150 samples, *Fusarium* made up a high proportion (Figure 5). Amplicon sequencing within the TEF1α gene region was used to resolve the *Fusarium* taxonomy (Figure 6).

While the ITS2 data clearly showed an abundance of *Fusarium*, to provide further evidence of correct taxonomy at the species level, the TEF1α taxonomic assignments (Figure 6) were cross-compared between the results for both gene regions. *F. acutatum*, *F. oxysporum*, and *F. solani* were common across both analyses. Additionally, ASVs of TEF1α were aligned successfully (>99% similar in this TEF region) with *Fusarium*-ID v3.0 for *F. acuminatum*, *F. avenaceum*, *F. brachygibbosum*, *F. brevicaudatum*, *F. clavum*, *F. equiseti*, *F. flagelliforme*, *F. graminearum*, *F. iranicum*, *F. keratoplasticum*, *F. nelsonii*, *F. neocosmosporiellum*, *F. oxysporum*, *F. redolens*, *F. sambucinum*, *F. solani*, *F. vanettenii*, and *Fusarium* sp. (undescribed), for which details of their NRRL (ARS culture collection number) and species complex identities are detailed in Table 8. The *F. acutatum*, *F culmorum*, *F. falciforme*, *F. napiforme*, *F. sacchari,* and *F. torulosum* taxonomic assignments from NCBI were not confirmed with the sequence in *Fusarium*-ID and were, thus, grouped under *Fusarium* sp. (in doubt) along with other unconfirmed taxa, while *F. ananatum* and *F. dimerum* were not present in the *Fusarium*-ID database so were retained in the bar chart. The sequence labelled *F*. *heterosporum* was more closely aligned with *F. graminum* (NRRL_20692) in the *Fusarium*-ID database.

**Figure 7:**
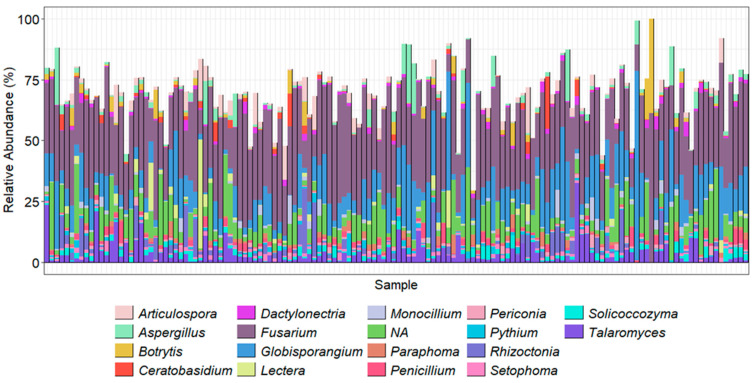
Using ITS1Oo primers, the top 20 most abundant genera present in each sample. Taxonomy was assigned using the ITSOo gene region, Vsearch assignment method, and a database generated from GenBank.

The ITSOo metabarcoding also amplified other fungal taxa. While this again demonstrated the dominance of *Fusarium* in the samples, it also helped highlight the Oomycetes present in the samples. The results demonstrated that *Globisporangium* spp. and *Pythium* spp. were abundant pathogens in the soil samples, making up nearly 10% and 8% of the top 20 most abundant genera, respectively (Figure 7).

## 4. Discussion

### 4.1. Soilborne Pathogens from Survey qPCR Study

The qPCR testing of the soil samples collected throughout the main faba bean and pulse growing regions of Ethiopia revealed a wide range of soilborne pathogens, many of which co-occur in pathogen complexes. These included root fungal pathogens such as *M. phaseolina*, *F. redolens*, and *R. solani* and Oomycetes such as *A. euteiches*, *Pythium* clades F and I, and *Ph. megasperma*. We believe this study is the first report of *F. redolens*, *A. euteiches*, *Ph. megasperma*, and *T. basicola* in Ethiopia. It is also the first time that five anastomosis groups of *R. solani*, namely AG2.1, AG2.2, AG3, AG4, and AG5, of which AG2.2 and AG4 were most prevalent, have been reported in Ethiopia. The foliar fungal pathogens *Botrytis cinerea*, *B. fabae. Didymella pinodes*, and *Phoma pinodella* were detected with high incidence rates, as was the nematode *Pratylenchus thornei*. Across all seven geographical zones of Ethiopia, most of the pathogens associated with faba bean and pulses were found to coexist to varying degrees, with root disease and multiple root rot pathogens found in each surveyed farm. Often, other foliar pathogens at varying population levels and frequencies also occurred. The root rot severity and distribution of these soilborne pathogens not only varied significantly between geographical zones but across soil types and with the soil pH. However, there were generally overall similar trends in terms of pathogen incidence across the three different host species (faba bean, field pea, lentil).

*Macrophomina phaseolina*, *Pythium* clades F and I, *F. redolens*, *R. solani,* and *A. euteiches* had the highest incidence rates, indicating they likely are significant root-rot-causing pathogens of faba bean, and likely also of field pea and lentil, in Ethiopia. Previously, Abreham [9] and Eshetu et al. [42] only reported *F. oxysporum* and *R. solani* as primary causes of faba bean root rot. In this study, the pathogens not only varied in terms of their incidence but also in terms of their abundance, as indicated by the DNA levels in soil. *D. pinodes*, *P. pinodella*, *R. solani* AG2.2, and AG4 recorded the highest DNA concentrations. Similarly, Lievens et al. [43] also showed that the *R. solani* complex is often contained in high amounts in mixed soil samples. Of the 14 defined groups of *R. solani* anastomosis that Carling et al. [44] reported, four of these were found in Ethiopia. However, the most aggressive anastomosis group (AG8) described by Hane et al. [45], a serious disease outside of Ethiopia, was not recorded. The highest incidence rate was for *Pythium* clade I (149/150 sites), followed by *M. phaseolina* (146/150 sites) and *Pythium* clade F (126/150 sites). Lievens et al. [43] also made similar observations for *Pythium* spp., which were virtually present in all cultivated soils and could be detected easily using DNA quantification. The high concentrations of *R. solani* and *M. phaseolina* are likely fostered by their presence in the soil in the form of mycelium and their known ability to persist for long periods of time. The time of sample collection can also affect the molecular quantification of the pathogens; for example, *Pythium* spp. do not thrive in dry soil and form dormant spores that may yield less DNA than in the vegetative state [46].

There was a strong, consistent influence of the geographical zone on the pathogen populations, likely a consequence of variations in soil texture, weather, fertility, and cropping history. This highlights the need to carefully consider zonal influences on the incidence of root rot pathogens in the soil when developing soil management plans to maximize the sustainable production of faba bean crops. It is perhaps not surprising that the soilborne pathogen populations were greatest in West Shoa and North Shoa for Amhara and North Shoa and West Arsi for Oromia, where the mean temperatures range from 7.9 to 23.5 °C and high rainfall in the range of 772–1123 mm occurs, with high rainfall being known to foster the highest populations of root rot pathogen colonization [47]. Similarly, Mwang’ombe et al. [48] found that areas of Kenya with high soil moisture contents had higher populations of pathogens that promoted the development of severe root rot of the common bean. Additionally, Naseri [49] noted that *Fusarium* spp. were a major cause of root rot under humid, acidic, and inadequately fertilized soil conditions, while Sun et al. [50] found that high soil carbon and moisture levels encouraged soilborne fungal populations and their growth. In another study in Kenya, *F. oxysporum* was found to be more widely distributed in humic nitisol, rhodic ferralsol, and vertisol soils than *F. proliferatum* and *F. incarnatum,* which were particularly more abundant in vertisol soils [51]. In this study, the incidence rates of *A. euteiches* were significantly higher in West Shoa and West Arsi, which can likely be attributed to the heavy vertisol soils and high moisture content due to water logging, as was the case for *Pythium* clade I in West Shoa. As zoospores of *A. euteiches* and *Pythium* spp. swim freely [52] and as Oomycetes are known to be particularly serious pathogens in heavier soils such as clay soils [53] and in waterlogged soils [54], it is not surprising *A. euteiches* and *Pythium* spp. were so prevalent in these heavy and frequently waterlogged vertisol soils. Further, the high retention rates of highly decomposed organic matter in clay soils provides an abundance of nutrients for *Pythium* spp. and *Aphanomyces* spp. [55].

In addition to the water-holding capacity and soil carbon level, the soilborne fungal diversity is also known to be influenced by the soil pH [56], the availability of a suitable host (especially pulses), and the intensity of cultivation of a host. Rouphael et al. [57] confirmed that the fungal population diversity is controlled by numerous biotic (plants and other organisms) and abiotic (soil pH, moisture, salinity, structure, and temperature) factors. The root rot severity was significantly lower in the Bale zone, likely because of the humic soils providing an ideal environment for competition between pathogenic and non-pathogenic organisms over soil nutrients, thereby reducing the disease incidence [58].

In the current study, the populations of *A. euteiches*, *F. redolens*, *Ph. megasperma*, *Pythium* clade F, *Pythium* clade I, *S. sclerotiorum*/*S. minor*, *T. basicola*, *V. dahliae*, and *R. solani* were lowest in mixed and nitisol soils, while for *R. solani* AG2.1 and AG5 and *M. phaseolina*, there were no differences across the three soil types. The study by Naseri [50] found the highest populations of *Macrophomina*, *Pythium*, *Rhizoctonia*, and *Fusarium* spp. in fine loamy sand soil followed by sandy clay soil. Gill et al. [59] demonstrated how quickly *R. solani* grows in sandy soils lacking in nutrients. Yaquelin et al. [60] also noted *R. solani* was more prevalent in calcisol than in ferralsol or vertisol soils.

### 4.2. Soilborne Pathogens from Metabarcoding Study

Analyses of the fungal community structure in the 150 faba bean field soils using DNA metabarcoding for general fungi and *Fusarium* species with ITS2 and TEF1α primers successfully highlighted *F. oxysporum* as the most abundant soilborne fungal pathogen, as well as *F. ananatum*, *F. brachygibbosum*, *F. brevicaudatum*, *F. clavum*, *F. flagelliforme*, *F. keratoplasticum*, *F. napiforme*, *F. nelsonii*, *F. neocosmosporiellum*, *F. torulosum*, and *F. vanettenii,* as their first reports of occurrence for Ethiopia. The ITS1Oo primer confirmed the Pythiaceae family as the most prevalent of all Oomycetes. Metabarcoding, involving the simultaneous amplification and sequencing of barcode sequences directly from environmental samples, proved to be a powerful technique to explore the diversity of microorganisms, including slow-competing or uncultivable taxa, in Ethiopian pulse soils [61].

Using the ITS2 primer, Pleosporales and Hypocreales were the most prevalent fungal groups among the top 30 orders on farms cropping faba bean, both in terms of the relative number of sequences in each sample and the number of samples in which they were found. It is interesting to note that the Hypocreales ASVs generally made up a higher proportions of the total reads in the sample than *Gnomonia* and *Diaporthe*, suggesting that Hypocreales also dominate in terms of the mycelial biomass. In terms of both the relative abundance and number of occurrences in the 150 soil samples, the majority of the frequent ASVs, as determined by the taxonomic assignment methods used here, exhibited affinity with well-known soilborne pathogens belonging to the orders Sordariales, Ophiostoma, Tremellales Cantharellales, and Helotiales.

In Ethiopia, many reports suggest that root rot of faba bean crops is mainly caused by *Fusarium* spp. [10,43]. However, these earlier analyses of *Fusarium* spp. composition in field samples were performed using the morphological identification or species-specific PCR-based methods [62]. Those PCR-based methods are reliable methods to detect and quantify various *Fusarium* spp. in field samples [63] but constitute an a priori approach restricted to known and specific targets. In the current study, the metabarcoding-based protocol for evaluating *Fusarium* spp. diversity on faba crops allowed more accurate species discrimination, as the TEF1α gene, a component of eukaryotic cells’ protein translation machinery, is effective in differentiating related *Fusarium* spp. [64,65]. The reason for the taxonomic assignment discrepancies we identified could be due to an inadequate representation of diversity in *Fusarium*-ID v3.0 or inaccurate sequences in NCBI. Despite this, we believe that the data generated here provide quite a comprehensive look at the *Fusarium* species that were present. The data enabled the detection of multiple different *Fusarium* spp. in the 150 soil samples, of which we could identify 16 species with relative confidence. *F*. *oxysporum* and *F*. *avenaceum* were the dominant species, followed by *F. solani.* These three species are common *Fusarium* spp. previously recovered from Ethiopia pulse crops [9,10,38]. Further, Alford et al. [65] highlighted the occurrence of *F. oxysporum* and other wilt pathogens from chickpea roots and stems across the major chickpea geographic area of Ethiopia. Importantly, as indicated above, to the best of our knowledge, we believe our study constitutes the first report for Ethiopia of *F. ananatum*, *F. brachygibbosum*, *F. brevicaudatum*, *F. clavum*, *F. flagelliforme*, *F. keratoplasticum*, *F. napiforme*, *F. nelsonii*, *F. neocosmosporiellum*, *F. torulosum*, *and F. vanettenii*. Although not highlighted here, these TEF1α primers also effectively amplified taxa in other genera, such as *Paraphoma*, *Purpureocillium*, *Macrophomina*, *Cladophialophora*, *Trichoderma,* and *Metarhizium*.

The ITS1Oo primer, a modification of the original ITSO [29], was chosen due it its greater specificity for Oomycetes than the previously developed ITSO, ITS6, and ITS7 primers, as it has an extra 3′ terminal adenine compared to ITS-O [30] (Riit et al. 2016). The predominant assigned Oomycete ASVs were *Globisporangium* spp. and *Pythium* spp. Unlike the earlier Oomycete studies by Nicolaisen et al. [66], *Globisporangium* spp. were predominant in the soil samples, accounting for almost 10% of the total Oomycete reads, followed by *Pythium* spp. Specifically, *Globisporangium ultimum* and *G. heterothallicum* were both identified in this study as relatively abundant species in these samples.

## 5. Conclusions

The root disease severity surveys across faba bean fields highlighted the widespread occurrence of serious root rot disease in Ethiopia. The application of species-specific qPCR testing and HTS metabarcoding studies on associated soil samples enabled the first comprehensive identification of soilborne pathogens in Ethiopian faba bean and pulses soils and their association with faba bean root disease. Previous attempts at the identification of soilborne pathogens have frequently relied upon cultural and morphological characteristics, providing at best a very poor representation of the actual pathogen components involved in root disease. This is especially true in situations where clearly multiple pathogen complexes are associated with these root disease disorders of faba bean, pea, and lentil in Ethiopia, limiting (or compromising) the management of such soilborne disease complexes [16,17,19]. The ability to reliably and accurately define the pathogens involved in soilborne disease complexes is foundational to being able to predict and manage them [20], and the current study has provided this foundation. As fungicides are unlikely to be effective in situations where multiple soilborne pathogens operating as disease complexes [19], the approach needed for the effective management of faba bean, pea, and lentil root rot diseases in Ethiopia involves screening to determine host resistances and ‘in-field tolerances’ and the integration of these processes within an IDM framework. While the widespread occurrence of the most prevalent pathogens has now been defined, there remains a need for further research to determine their respective roles and significance, both individually and in pathogen complexes, in causing severe root rot disease, not only of faba bean but also field pea and lentil.

## Figures and Tables

**Table 1:** Climatic, elevation, and soil conditions within each of the different geographical zones where samples were collected.

Study Zone	Altitude (m)	Annual Rainfall (mm)	Average Annual Temperature Range (°C)	Root Zone Moisture (%)	Soil Surface Moisture (%)	Crop Type		
						Faba Bean	Field Pea	Lentil
South Wollo	2605–2793	889–939	8.7–21.9	57–88	52–85	18	-	-
North Shoa (AM)	2462–3210	772–927	9.2–21.8	62–89	59–87	15	6	-
North Shoa (OR)	2510–3097	772–1038	7.9–19.0	64–91	60–88	20	1	-
West Shoa	2135–2699	847–1123	10.5–23.5	69–94	67–90	19	4	-
Arsi	2366–2976	587–799	8.1–21.2	52–77	50–73	20	-	-
West Arsi	2215–2840	770–851	9.6–21.5	55–85	53–82	18	-	-
Bale	2359–3334	666–858	8.78–21.4	50–80	49–76	16	7	6

**Table 2:** Quantitative PCR (qPCR) soil test results of root fungal and Oomycete soilborne pathogens, fungal leaf pathogens, and nematode species, in terms of their incidence rates out of the 150 samples (consisting of 125 that were associated with faba bean crops, 18 with field pea crops, and 6 with lentil crops) and their average (Ave) and highest DNA concentrations across the 150 samples.

Pathogen	* Ethiopian New Record	Unit	Faba Bean (126 Sites)			Pea (18 Sites)			**Lentil (6 Sites)**		
			Incidence (No./126)	Ave DNA	Highest DNA	Incidence (No./18)	Ave DNA	Highest DNA	**Incidence** **(No./6)**	**Ave** **DNA**	**Highest** **DNA**
**Root pathogens**											
*Aphanomyces euteiches*	*	pg DNA/g sample	44	6	165	5	10	140	2	19	102
*Fusarium redolens*	*	Ct value	66	35	44	8	34	39	3	35	37
*Macrophomina phaseolina*	*	kDNA copies/g sample	123	47	316	16	39	112	6	70	224
*Phytophthora medicaginis*	*	kDNA copies/g sample	1	0.002	0.21	0	0	0	0	0	0
*Phytophthora megasperma*	*	kDNA copies/g sample	36	0.6	6.13	5	2	16	2	1	3
*Pythium clade F*		pg DNA/g sample	108	61	417	13	38	121	4	52	157
*Pythium clade I*		pg DNA/g sample	124	135	1651	18	115	656	6	120	293
*S. sclerotiorum*/*S. minor*		kDNA copies/g sample	32	83	2562	2	76	1360	1	3	21
*Thielaviopsis basicola*	*	kDNA copies/g sample	22	2.3	72	1	1	13	0	0	0
*Verticillium dahliae*		pg DNA/g sample	27	0.9	41	2	0	1	2	1	3
*Rhizoctonia solani AG2.1*	*	pg DNA/g sample	63	32	1039	11	32	225	4	10	34
*Rhizoctonia solani AG2.2*	*	pg DNA/g sample	44	240	18,390	5	227	2489	1	11	67
*Rhizoctonia solani AG3*	*	pg DNA/g sample	16	18	728	3	2	31	1	0	2
*Rhizoctonia solani AG4*	*	pg DNA/g sample	39	119	4221	7	1015	15,244	3	37	165
*Rhizoctonia solani AG5*	*	pg DNA/g sample	6	18	1971	0	0	0	0	0	0
**Leaf pathogens**											
*Ascochyta fabae*		kDNA copies/g sample	53	176	12,143	5	31	349	3	233	856
*Ascochyta lentis*		kDNA copies/g sample	2	0.05	6.08	0	0	0	0	0	0
*Botrytis cinerea*		kDNA copies/g sample	100	14	440	13	2	13	4	4	20
*Botrytis fabae*		kDNA copies/g sample	120	330	4500	18	206	1663	6	75	316
*Didymella pinodes/Phoma pinodella*		pg DNA/g sample	110	1051	19,256	16	1151	15,162	5	370	1450
*Phoma rabiei*		kDNA copies/g sample	2	0.01	0.61	0	0	0	0	0	0
**Nematodes**											
*Meloidogyne javanica/incognita*/*arenaria*		pg DNA/g sample	6	23	1791	0	0	0	0	0	0
*Pratylenchus neglectus*		nematodes/g soil	4	0.04	4.06	0	0	0	0	0	0
*Pratylenchus penetrans*		nematodes/g soil	4	0.06	3.34	0	0	0	0	0	0
*Pratylenchus quasitereoides*		nematodes/g soil	1	0	0.02	0	0	0	0	0	0
*Pratylenchus thornei*		nematodes/g soil	75	1	31	13	6	93	5	4	14

Note: The different units arose as newer tests were developed with calibration standards using synthetic oligonucleotides (reported as kDNA copies/gram sample) instead of genomic DNA (reported as pg DNA/gram sample). The nematode tests were calibrated to convert the amount of DNA measured via qPCR into the equivalent number of nematodes. * denotes pathogen is a new first record for Ethopia.

**Table 3:** Soil microbiota DNA quantification of individual pathogens in relation to root rot Percentage Severity Index (PSI) scores.

**PSI**	**No.**	***A. euteiches***			***M. phaseolina***			***Ph. megasperma***			***Pythium* clade F **			***Pythium* clade I **			***T. basicola***			***V. dahliae***		
		**Ave**	**Min**	**Max**	**Ave**	**Min**	**Max**	**Ave**	**Min**	**Max**	**Ave**	**Min**	**Max**	**Ave**	**Min**	**Max**	**Ave**	**Min**	**Max**	**Ave**	**Min**	**Max**
1–5	45	6	0	165	38	0	169	0.5	0	4	57	0	368	93	0	587	0.6	0	11	0	0	3
6–15	35	4	0	49	57	1	316	0.9	0	6	63	0	319	109	0	770	3.4	0	72	0	0	4
16–25	22	9	0	87	63	0	216	0.3	0	2	50	0	143	149	2	810	4.5	0	69	1	0	7
26–50	17	7	0	37	38	2	110	0.7	0	6	43	0	290	296	5	1651	0.6	0	8	1	0	10
>50	6	10	0	37	25	2	100	0.3	0	1	173	0	417	90	10	201	5.9	0	33	5	0	41
		***R. solani AG2-1***			***R. solani AG2-2***			***R. solani AG3***			***R. solani AG4***			***R. solani AG5***			***A. fabae***			***B. cinerea***		
**PSI**	**No.**	**Ave**	**Min**	**Max**	**Ave**	**Min**	**Max**	**Ave**	**Min**	**Max**	**Ave**	**Min**	**Max**	**Ave**	**Min**	**Max**	**Ave**	**Min**	**Max**	**Ave**	**Min**	**Max**
1–5	45	20	0	225	175	0	3095	6	0	226	118	0	4017	38	0	1971	69	0	881	7	0	104
6–15	35	22	0	143	101	0	1343	55	0	728	325	0	4221	3	0	78	10	0	225	41	0	440
16–25	22	50	0	1039	563	0	18,390	3	0	87	473	0	15,244	6	0	2226	358	0	12,143	4	0	40
26–50	17	38	0	697	9	0	143	9	0	101	3	0	39	0	0	0	289	0	4355	6	0	63
>50	6	22	0	127	8	0	67	13	0	100	19	0	165	0	0	0	61	0	518	5	0	21
		***P. rabiei***			***M. javanica++***			***P. neglectus***			***P. thornei***			***B. fabae***			***D. pinodes/pinodella***			***P. penetrans***		
**PSI**	**No.**	**Ave**	**Min**	**Max**	**Ave**	**Min**	**Max**	**Ave**	**Min**	**Max**	**Ave**	**Min**	**Max**	**Ave**	**Min**	**Max**	**Ave**	**Min**	**Max**	**Ave**	**Min**	**Max**
1–5	45	0	0	0	0	0	3	0	0	4	0	0	5	259	0	3831	3010	0	19,256	0	0	0
6–15	35	0	0	0	1	0	0	0	0	0	2	0	31	416	0	4500	1457	0	16,631	0	0	3
16–25	22	0	0	0	0	0	396	0	0	1	3	0	93	255	0	4116	1103	0	15,162	0	0	0
26–50	17	0	0	0	0	0	1791	0	0	0	1	0	14	145	0	754	744	0	6884	0	0	0
>50	6	0	0	0	0	0	0	0	0	0	2	0	9	859	0	3739	223	0	675	0	0	3

PSI = Percentage Severity Index; No. = number of samples within each PSI scale range; Ave = average DNA concentration, Min = minimum DNA concentration; Max = maximum DNA concentration. Note: For DNA concentration units, please refer to Table 2.

**Table 4:** An analysis of soilborne pathogen abundance levels based on DNA concentrations as affected by the soil type and geographical zone and their interaction.

Source Variation		Significances in Relation to						
	pH	PSI	*A. euteiches*	*F. redolens*	*M. phaseolina*	*Ph. megasperma*	*Pythium clade F*	*Pythium clade I.*
Soil type	***	***	***	*	*ns*	**	*ns*	*
Zone	***	**	*ns*	*ns*	*ns*	*	*ns*	**
Soil type x Zone	***	*ns*	*ns*	*ns*	*ns*	*ns*	*ns*	***
	***S. sclerotiorum/minor.***	***T. basicola***	***V. dahliae***	***R. solani AG2.1***	***B. fabae***	***R. solani AG2.2***	***R. solani AG3***	***R. solani AG4***
Soil type	***	**	*ns*	*ns*	*ns*	**	*ns*	*ns*
Zone	*ns*	*ns*	***	*ns*	**	*ns*	**	*ns*
Soil type x Zone	*ns*	**	*ns*	*ns*	**	*ns*	*ns*	*ns*
	***R. solani AG5***	***D. pinodes+***	***P. rabiei***	***M. javanica++***	***P. neglectus***	***P. penetrans***	***P. quasitereoides***	***P. thornei***
Soil type	*ns*	**	*ns*	***	*ns*	*ns*	*ns*	*ns*
Zone	*ns*	*	*ns*	*ns*	*ns*	*ns*	*ns*	*
Soil type x Zone	*ns*	*ns*	*ns*	*ns*	*ns*	*ns*	*ns*	*ns*

PSI = Percentage Severity Index; *** = significant at *p* ≤ 0.001; ** = significant at *p* ≤ 0.01; * = significant at *p* ≤ 0.05; *ns* = not significant.

**Table 5:** Distribution patterns of pH levels, soilborne disease severity levels, and soilborne pathogens, as quantified using the commercial quantitative PCR (qPCR) soil testing service, across the different geographical zones. Survey data and results of quantitative PCR (qPCR) soil tests were analyzed using R-software version number 4.0.2 [32]. DNA concentrations of the respective pathogen, soil type, soil pH, agroecology region, and PSI values for root disease were subjected to an analysis of variance (ANOVA), and the Tukey test for the least significant difference (LSD) was used for mean separation at the 5% level of significance.

Source of Variation	pH	PSI	Pathogens										
**Geographical Zone**			***A. eut***	***F. red.***	***M. ph***	***P. meg***	***Py. F***	***Py. I***	***S. scl***	***T. bas***	***V. dha***	***R.A2.1***	***R.A2.2***
Arsi	4.9 ^bc^	10.7 ^bc^	2.8 ^ab^	19.8 ^a^	82.5 ^a^	1.1 ^a^	48.8 ^ab^	93.1 ^bc^	71.5 ^a^	0.5 ^c^	0.1 ^b^	42.5 ^a^	83.9 ^a^
Bale	5.7 ^ab^	5.1 ^c^	0.6 ^b^	10.9 ^ab^	90.9 ^a^	0.6 ^ab^	37.9 ^ab^	64.6 ^bc^	0 ^a^	0.4 ^c^	0 ^b^	8.0 ^a^	180.3 ^a^
North Shoa Amhara	5.9 ^ab^	14.4 ^ab^	6.2 ^ab^	17.0 ^a^	35.5 ^ab^	0.5 ^ab^	51.9 ^ab^	66.0 ^bc^	32.9 ^a^	0.04 ^c^	0.8 ^b^	3.1 ^a^	42.3 ^a^
North Shoa Oromia	4.2 ^cd^	10.4 ^bc^	3.0 ^ab^	13.3 ^ab^	39.2 ^ab^	0.3 ^b^	40.5 ^ab^	88.5 ^bc^	118.2 ^a^	6.2 ^a^	0.2 ^b^	25.1 ^a^	119.7 ^a^
South Wollo	3.5 ^d^	5.7 ^c^	3.4 ^ab^	4.9 ^b^	19.9 ^b^	0.2 ^b^	22.5 ^b^	30.5 ^c^	95.7 ^a^	0.04 ^c^	0.4 ^b^	7.5 ^a^	167.1 ^a^
West Arsi	4.2 ^cd^	15.0 ^ab^	9.9 ^a^	15.4 ^ab^	15.0 ^b^	0.2 ^b^	62.2 ^a^	111.0 ^b^	16.6 ^a^	3.0 ^b^	0.6 ^b^	15.8 ^a^	119.0 ^a^
West Shoa	6.4 ^a^	20.5 ^a^	9.2 ^a^	19.8 ^a^	48.1 ^ab^	1.0 ^a^	46.2 ^ab^	207.0 ^a^	101.7 ^a^	2.0 ^bc^	3.7 ^a^	48.6 ^a^	16.6 ^a^
LSD	1.12	8.08	7.22	11.04	56.95	0.63	36.60	78.34	123.15	2.41	1.66	52.42	208.13
**Geographical Zone**	***R.AG3***	***R.AG4***	***R.AG5***	***A. fab***	***B. cin***	***B. fab***	***D. pin***	***P. rab***	***M. jav***	***P. neg***	***P. pen***	***P. qua***	***P. tho***
Arsi	19.9 ^abc^	39.3 ^a^	0 ^a^	2.3 ^b^	2.6 ^b^	126.9 ^c^	277.6 ^c^	0 ^a^	0 ^b^	0.01 ^a^	0.09 ^a^	0 ^a^	2.3 ^a^
Bale	12.9 ^bcd^	47.3 ^a^	0 ^a^	6.7 ^b^	3.1 ^b^	118.1 ^c^	430.7 ^bc^	0 ^a^	0.1 ^b^	0 ^a^	0 ^a^	0 ^a^	0.11 ^b^
North Shoa Amhara	6.9 ^cd^	7.4 ^a^	0 ^a^	454.6 ^a^	9.7 ^ab^	108.3 ^c^	1142.3 ^ab^	0 ^a^	0.4 ^b^	0.02 ^a^	0 ^a^	0 ^a^	0.16 ^b^
North Shoa Oromia	38.1 ^a^	104.4 ^a^	8.5 ^a^	221.1 ^ab^	13.4 ^ab^	247.3 ^bc^	1166.9 ^ab^	0 ^a^	0 ^b^	0 ^a^	0.01 ^a^	0 ^a^	0.5 ^b^
South Wollo	0.1 ^d^	151.4 ^a^	0 ^a^	6.1 ^b^	4.9 ^ab^	405.4 ^ab^	392.9 ^bc^	0 ^a^	0 ^b^	0.15 ^a^	0 ^a^	0.1 ^a^	0.17 ^b^
West Arsi	30.7 ^ab^	164.2 ^a^	3.0 ^a^	8.8 ^b^	11.3 ^ab^	386.6 ^ab^	294.8 ^c^	0 ^a^	0.1 ^b^	0.01 ^a^	0 ^a^	0 ^a^	0.24 ^b^
West Shoa	17.5 ^bcd^	8.3 ^a^	0.5 ^a^	62.2 ^ab^	25.2 ^a^	459.3 ^a^	1307.3 ^a^	0.01 ^a^	165.0 ^a^	0 ^a^	0.13 ^a^	0 ^a^	1.3 ^ab^
LSD	19.69	193.12	9.02	402.92	22.10	202.87	845.12	0.01	61.93	0.16	0.16	0.00	1.44

PSI = Percentage Severity Index; *A. eut* = *Aphanomyces euteiches*; *F. red* = *Fusarium redolens*; *M. pha* = *Macrophomina phaseolina*; *P. meg* = *Phytophthora megasperma*; *Py. F* = *Pythium* clade F; *Py. I* = *Pythium* clade I; *S. scl* = *Sclerotinia sclerotiorum*/*S. minor*; *T. bas* = *Thielaviopsis basicola*; *V. dha* = *Verticillium dahliae*; *R.AG2-1* = *Rhizoctonia solani* AG2.1; *R.AG2.2* = *Rhizoctonia solani* AG2.2; *R.AG3* = *Rhizoctonia solani* AG3; *R.AG4* = *Rhizoctonia solani* AG4; *R.AG5* = *Rhizoctonia solani* AG5; *A. fab* = *Ascochyta fabae*; *B. cin* = *Botrytis cinerea*; *B. fab* = *Botrytis fabae*; *D. pin* = *Didymella pinodes*/*Phoma pinodella*; *P. rab* = *Phoma rabiei*; *M. jav* = *Meloidogyne* *javanica*/*incognita*/*arenaria*; *P. neg* = *Pratylenchus neglectus*; *P. pen* = *Pratylenchus penetrans*; *P. qua* = *Pratylenchus quasitereoides*; *P. tho* = *Pratylenchus thornei*. Means with different superscript letters within the same column are significantly different at *p* ≤ 0.05, LSD.

**Table 6:** Distributions pattern of soilborne pathogens quantified via qPCR, using the commercial quantitative PCR (qPCR) soil testing service, in different soil types. Results were analyzed using R-software version number 4.0.2 [32]. DNA concentrations of the respective pathogen, soil type, soil pH, and PSI values for root disease were subjected to an analysis of variance (ANOVA), and the Tukey test for the least significant difference (LSD) was used for mean separation at the 5% level of significance.

Soil Type	pH	PSI	*A. eut*	*F. red*	*M. pha*	*P. meg*	*Py. F*	*Py. I*	*S. scl*	*T. bas*	*V. dha*	*R.AG2.1*	*R.AG2.2*
Mixed	5.1 ^b^	10.0 ^b^	2.8 ^b^	11.1 ^b^	32.9 ^a^	0.4 ^b^	28.4 ^b^	63.4 ^b^	18.7 ^b^	2.1 ^a^	0.2 ^b^	21.9 ^a^	33.9 ^b^
Nitisol	3.3 ^c^	6.5 ^b^	0.9 ^b^	11.8 ^b^	51.6 ^a^	0.3 ^b^	18.8 ^b^	83.8 ^b^	11.9 ^b^	0.1 ^b^	0.7 ^ab^	7.6 ^a^	45.9 ^b^
Vertisol	6.5 ^a^	18.5 ^a^	11.3 ^a^	20.5 ^a^	57.4 ^a^	0.9 ^a^	85.7 ^a^	135.9 ^a^	156.5 ^a^	3.0 ^a^	1.5 ^a^	35.1 ^a^	232.6 ^a^
LSD	0.7	5.3	4.7	7.2	37.3	0.4	24.0	51.3	80.6	1.6	1.1	34.3	136.2
	***R.AG3***	***R.AG4***	***R.AG5***	***A. fab***	***B. cin***	***B. fab***	***D. pin***	***P. rab***	***M. jav***	***P. neg***	***P. pen***	***P. qua***	***P. tho***
Mixed	4.2 ^b^	9.0 ^b^	0 ^a^	11.4 ^a^	6.1 ^a^	181.0 ^b^	556.6 ^b^	0 ^a^	0 ^b^	0.01 ^a^	0.05 a	0 a	0.5 ^ab^
Nitisol	9.8 ^b^	22.7 ^b^	0.2 ^a^	50.0 ^a^	6.7 ^a^	143.5 ^b^	323.6 ^b^	0 ^a^	0 ^b^	0 ^a^	0 a	0 a	0.2 ^b^
Vertisol	40.1 ^a^	192.1 ^a^	4.9 ^a^	265.2 ^a^	17.2 ^a^	469.3 ^a^	1268.0 ^a^	0 ^a^	70.9 ^a^	0.07 ^a^	0.05 a	0 a	1.3 ^a^
LSD	12.9	126.4	5.9	263.8	14.5	132.8	553.3	*ns*	40.5	0.11	0.10	0.00	0.95

PSI = Percentage Severity Index; *A. eut* = *Aphanomyces euteiches*; *F. red* = *Fusarium redolens*; *M. pha* = *Macrophomina phaseolina*; *P. meg* = *Phytophthora megasperma*; *Py. F* = *Pythium* clade F; *Py. I* = *Pythium* clade I, *S. scl* = *Sclerotinia sclerotiorum*/*S. minor*, *T. bas* = *Thielaviopsis basicola*, *V. dha* = *Verticillium dahliae*; *R.AG2.1* = *Rhizoctonia solani* AG2.1; *R.AG2.2* = *Rhizoctonia solani* AG2.2; *R.AG3* = *Rhizoctonia solani* AG3, *R.AG4* = *Rhizoctonia solani* AG4, *R.AG5* = *Rhizoctonia solani* AG5; *A. fab* = *Ascochyta fabae*; *B. cin* = *Botrytis cinerea*; *B. fab* = *Botrytis fabae*; *D. pin* = *Didymella pinodes*/*Phoma pinodella*; *P. rab* = *Phoma rabiei*; *M. jav* = *Meloidogyne javanica*/*incognita*/*arenaria*; *P. neg* = *Pratylenchus neglectus*; *P. pen* = *Pratylenchus penetrans*; *P. qua* = *Pratylenchus quasitereoides*; *P. tho* = *Pratylenchus thornei*. Means with different superscript letters within same column are significantly different at *p* ≤ 0.05, LSD. *ns* = not significantly different at *p* ≤ 0.05.

**Table 7:** Effect of soil drainage (raised beds) versus no drainage on the distribution of selected soilborne pathogens in vertisol and other soil types.

	Vertisol Soils			Other Soil Types			All
	No Drainage	Drainage	Overall	No Drainage	Drainage	Overall	
Number of sites	22	24	46	58	21	79	125
Root rot incidence (%)	25.8	10.7	17.9	15.6	14.7	15.3	16.3
Average readings							
*Aphanomyces euteiches*	4.4	6.1	5.3	8.6	2.5	7.0	6.4
*Macrophomina phaseolina*	32.8	46.2	39.8	53.7	44.8	51.4	47.1
*Pythium* clade F	90.5	50.8	69.8	57.5	51.6	56.0	61.0
*Pythium* clade I	166.7	69.3	115.9	171.0	75.8	145.7	134.7
*Rhizoctonia solani* AG2.1	19.1	28.3	23.9	44.6	14.8	36.7	32.0
*Rhizoctonia solani* AG2.2	162.3	44.0	100.6	405.2	86.5	320.5	239.6
*Rhizoctonia solani* AG3	7.1	46.9	27.9	14.9	4.3	12.1	17.9
*Rhizoctonia solani* AG4	254.6	118.1	183.4	91.6	56.4	82.3	119.5
*Rhizoctonia solani* AG5	0.0	9.6	5.0	1.6	93.9	26.1	18.4

**Table 8:** Isolates from *Fusarium*-ID v3.0 with >99% similarity to amplicon sequence variants (ASVs) generated with TEF1α primers.

Isolate ID	Species Complex	Species Name
NRRL_22278	*F. solani* Species Complex (FSSC) 11a	*F. vanettenii*
NRRL_22436	FSSC 8b	*F. neocosmosporiellum*
NRRL_32484/NRRL_32810/NRRL_32492	FSSC 5j, m, and c	*F. solani*
NRRL_22640	FSSC 2i	*F. keratoplasticum*
NRRL_25221	FFSC Asian	*Fusarium* sp. (undescribed)
NRRL_22901	*F. redolens* Species Complex (FRSC)	*F. redolens*
CBS_143611	*F. tricinctum* species complex (FTSC) 6	*F. iranicum*
F1529	FTSC 4	*F. avenaceum*
P454a	FTSC 2	*F. acuminatum*
FRC_R8227	*F. sambucinum* species complex (FSAMSC)	*F. sambucinum*
NRRL_28505/NRRL_29011/NRRL_26083	FSAMSC	*F. graminearum*
NRRL_66931	FSAMSC	*F. brachygibbosum*
NRRL_46743	FSAMSC	*Fusarium* sp. (undescribed)
NRRL_13338/NRRL_28505	*F. chlamydosporum* species complex (FCSC) 4a and b	*F. nelsonii*
NRRL_45997/NRRL_43623	*F. incarnatum-equiseti* species complex (FIESC) 5 a,b,e, and f	*F. clavum*
NRRL_36269	FIESC 12a, b, and c	*F. flagelliforme*
NRRL_43638	FIESC 6a	*F. brevicaudatum*
NRRL_36321/NRRL43636	FIESC 14a and c	*F. equiseti*
NRRL_22550/NRRL_22544/NRRL_36471/NRRL_26437/NRRL_20433/NRRL_36286/NRRL_26438/NRRL_28366/NRRL_22534/NRRL_26033	*F. oxysporum* species complex (FOSC)	*F. oxysporum*

NRRL = ARS culture collection number.

## Data Availability

Data will be made available upon reasonable request.

## Abbreviations

The following abbreviations are used in this manuscript:

PSI	Percentage Severity Index
AG	Anastomosis group
HTS	High-throughput sequencing
qPCR	Quantitative PCR
IDM	Integrated disease management
ASVs	Amplicon sequence variants
SARDI	South Australian Research Development Institute
NRRL	ARS culture collection number
ARS	Agricultural Research Service
NCBI	National Center for Biotechnology Information
